# The Prognostic Value of Red Blood Cell Distribution Width-to-Albumin Ratio (RAR) in Predicting Mortality and Severity in Acute Pancreatitis: A Systematic Review and Meta-Analysis

**DOI:** 10.7759/cureus.81279

**Published:** 2025-03-27

**Authors:** Helai Hussaini, Kinan Obeidat, Abdelaziz Maali Abusal, Olaniyi Fadeyi, Ihtisham Habib, Sandipkumar S Chaudhari, Calvin R Wei, Shamsha Hirani

**Affiliations:** 1 Internal Medicine, West Anaheim Medical Center, Anaheim, USA; 2 Internal Medicine, University of Texas Medical Branch at Galveston, Galveston, USA; 3 Internal Medicine, Hamad General Hospital, Doha, QAT; 4 Internal Medicine, Medical Teaching Institute, Lady Reading Hospital Peshawar, Peshawar, PAK; 5 Cardiothoracic Surgery, University of Alabama at Birmingham, Birmingham, USA; 6 Family Medicine, University of North Dakota School of Medicine and Health Sciences, Fargo, USA; 7 Research and Development, Shing Huei Group, Taipei, TWN; 8 Cardiology, Baqai Hospital, Karachi, PAK

**Keywords:** acute pancreatitis, biomarker, mortality, prognostic, red blood cell distribution width albumin ratio

## Abstract

Acute pancreatitis is a potentially life-threatening inflammatory condition with variable clinical presentations. Early risk stratification remains challenging despite existing scoring systems. The red blood cell distribution width-to-albumin ratio (RAR) has emerged as a potential prognostic biomarker in inflammatory conditions. This systematic review and meta-analysis aimed to evaluate the association between admission RAR and outcomes in acute pancreatitis. We conducted a comprehensive literature search and identified five retrospective studies meeting the inclusion criteria. Meta-analysis was performed to assess the relationship between RAR and mortality as well as disease severity in acute pancreatitis patients. Pooled analysis demonstrated that elevated RAR was significantly associated with increased mortality risk (risk ratio (RR): 2.11, 95% confidence interval (95% CI): 1.35-3.30) with moderate heterogeneity (I²: 46%). When comparing disease severity, mean RAR values were significantly lower in mild acute pancreatitis compared to severe cases (mean difference (MD): -1.78, 95% CI: -2.09 to -1.46), also with moderate heterogeneity (I²: 44%). This meta-analysis suggests that RAR, a simple and cost-effective biomarker available from routine blood tests, may serve as a valuable prognostic indicator for mortality and severity in acute pancreatitis. Despite having comparatively lower discriminative ability than conventional scoring systems, RAR offers advantages in terms of rapid assessment and cost efficiency. However, limitations include the small number of included studies, their retrospective nature, and heterogeneity in study settings. Further prospective studies are warranted to validate these findings.

## Introduction and background

Acute pancreatitis (AP) is a common and potentially life-threatening inflammatory condition of the pancreas, characterized by a highly variable clinical course ranging from mild self-limiting disease to severe organ dysfunction and high mortality [1]. Despite advancements in critical care and management strategies, predicting the prognosis of acute pancreatitis remains a challenge due to its complex pathophysiology and diverse clinical presentations [2]. Early risk stratification of patients with acute pancreatitis is crucial for timely intervention and improved clinical outcomes. Several scoring systems, such as the Ranson criteria, the Acute Physiology and Chronic Health Evaluation (APACHE) II score, and the Bedside Index for Severity in Acute Pancreatitis (BISAP), have been developed to predict acute pancreatitis severity and mortality [3]. However, these scoring systems have limitations, including the requirement for multiple laboratory and clinical parameters, delayed applicability, and variability in performance across different populations. Therefore, there is a need for simpler, readily available, and cost-effective biomarkers for early risk assessment in acute pancreatitis [4]. 

Recently, the red blood cell distribution width-to-albumin ratio (RDW/Alb) known as RAR has gained attention as a potential prognostic biomarker in various inflammatory and critical illnesses [5,6]. RDW, a measure of the variation in red blood cell size, is commonly used in the differential diagnosis of anemia but has also been associated with systemic inflammation, oxidative stress, and adverse outcomes in conditions such as sepsis, cardiovascular disease, and cancer [7]. On the other hand, serum albumin, a marker of nutritional and inflammatory status, is known to decrease in critically ill patients and has been linked to worse clinical outcomes in acute pancreatitis [8]. The RAR combines these two biomarkers and may provide a more comprehensive reflection of the inflammatory and nutritional state of patients with acute pancreatitis, thereby serving as a valuable tool for mortality prediction [9]. 

Emerging evidence suggests that an elevated RDW/Alb ratio at admission is associated with increased severity and mortality in patients with acute pancreatitis [10,11]. However, individual studies have limited sample size, and the overall prognostic significance of this biomarker remains unclear. A systematic synthesis of available evidence is necessary to determine the robustness of this association and its potential clinical utility. Individual studies have limited sample size; thus, the overall prognostic significance of this biomarker remains unclear. By pooling data from existing studies, our analysis provides a more comprehensive understanding of RAR's prognostic value in AP, which may aid in early risk stratification and clinical decision-making. Therefore, this systematic review and meta-analysis aim to evaluate the association between the RDW/Alb ratio at admission and all-cause mortality in patients with acute pancreatitis. By pooling data from existing studies, we seek to provide a more comprehensive understanding of the prognostic value of RDW/Alb in AP, which may aid in early risk stratification and clinical decision-making. 

## Review

Methodology 

Literature Search and Search Strategy 

We conducted a comprehensive literature search in multiple electronic databases, including PubMed, Embase, Web of Science, Cochrane Library, and Scopus, from inception to 25 February 2025. The search strategy combined terms related to acute pancreatitis (e.g., "acute pancreatitis," "pancreatitis," and "pancreatic inflammation") and red blood cell distribution width-to-albumin ratio (e.g., "red cell distribution width," "RDW," "albumin," "RDW/Alb ratio," and "RAR"). We also manually searched the reference lists of retrieved articles and relevant reviews to identify additional eligible studies. No language or publication date restrictions were applied. The search was performed by two authors independently. Any disagreement between two authors was resolved through discussion or consensus with a third author if required. 

Study Selection 

Two independent reviewers screened the titles and abstracts of all retrieved studies. Studies were included if they met the following criteria: (1) enrolled adult patients with acute pancreatitis; (2) measured RDW/Alb ratio at admission; (3) reported outcomes including mortality and/or disease severity; and (4) provided sufficient data to calculate effect estimates. Case reports, case series, review articles, abstracts from conferences, editorials, and animal research were not included. A third reviewer was consulted or discussed in order to settle disagreements. The Preferred Reporting Items for Systematic Reviews and Meta-Analyses (PRISMA) standards were adhered to during the research selection procedure. 

Data Extraction and Quality Assessment 

Using a standardized data extraction form, two investigators independently extracted the following information: first author, publication year, study location, study design, sample size, patient demographics, RAR cutoff values (if applicable), effect estimates with corresponding 95% confidence intervals, and adjustment for confounders. When relevant data were not directly available, we contacted the corresponding authors or calculated values from the provided information where possible. 

The Newcastle-Ottawa Scale (NOS) for observational studies was used to evaluate the quality of the included research. This scale assesses research according to exposure or result determination, group comparability, and study group selection. High-quality studies had a NOS value of at least 7, moderate-quality studies had scores between 5 and 6, and low-quality studies had ratings less than 5. The quality of the work was evaluated independently by two reviewers, and disagreements were settled by consensus. 

Data Analysis 

Statistical analyses were performed using Review Manager (RevMan, version 5.4, Cochrane Collaboration, London, UK). For binary outcomes, we calculated risk ratios (RRs) with 95% confidence intervals (CIs). Heterogeneity among studies was assessed using the I² statistic, with values of 25%, 50%, and 75% representing low, moderate, and high heterogeneity, respectively. We used random-effects models for meta-analysis irrespective of the degree of heterogeneity among the study results to deal with variation among study results. A two-sided p-value <0.05 was considered statistically significant for all analyses. We were not able to perform publication bias as the number of included studies was less than 10.

Results 

**Figure 1:**
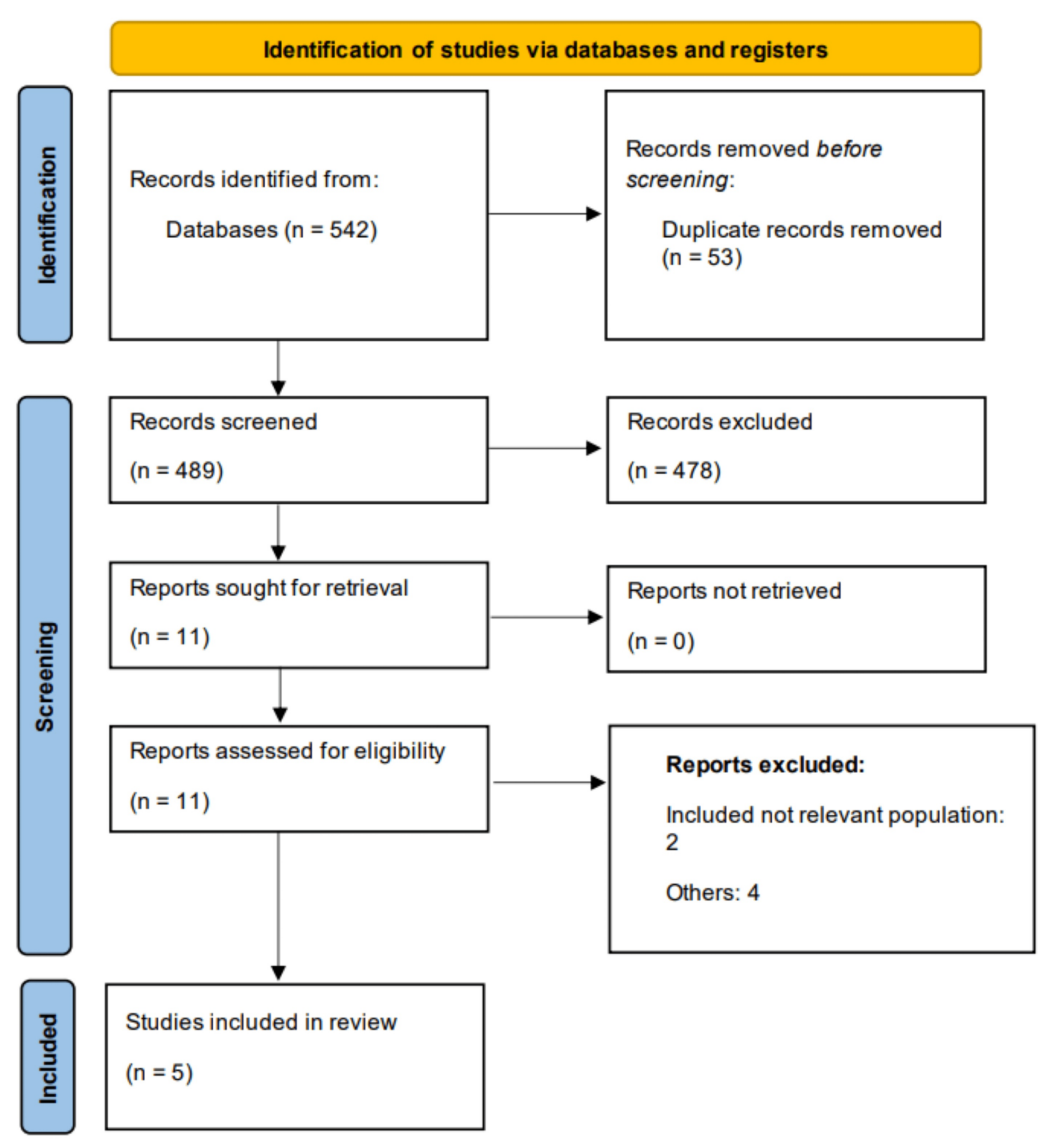
PRISMA flowchart of study selection. PRISMA: Preferred Reporting Items for Systematic Reviews and Meta-Analyses.

The initial database search identified 542 records. After eliminating 53 duplicates, 489 titles and abstracts were screened for relevance. Of these, 478 studies were excluded based on predefined eligibility criteria, resulting in 11 full-text articles selected for detailed evaluation. After reviewing the full texts, six studies were excluded. Ultimately, five studies met the inclusion criteria and were incorporated into the systematic review and meta-analysis. The study selection process is depicted in the PRISMA flow diagram (Figure 1). Table 1 summarizes the characteristics of the included studies, all of which were retrospective in design. Table 2 shows a quality assessment of the included studies.

**Table 1:** Characteristics of included studies. NR: not reported.

Author	Year	Study design	Place	Follow-up days	Sample size	Age (years)
Acehan et al. [12]	2024	Retrospective	Turkey	18 Months	365	57.7
Chen et al. [13]	2025	Retrospective	China	One year	648	58
Donmez et al. [9]	2022	Retrospective	Turkey	In hospital	166	62
Wang et al. [10]	2022	Retrospective	China	NR	301	48.5
Wu et al. [11]	2025	Retrospective	China	28 Days	931	58.3

**Table 2:** Quality assessment of included studies.

Author	Selection	Comparison	Assessment	Overall
Acehan et al. [12]	3	2	2	Good
Chen et al. [13]	4	2	1	Good
Donmez et al. [9]	3	1	2	Fair
Wang et al. [10]	3	2	2	Good
Wu et al. [11]	4	2	2	Good

Meta-Analysis of Outcomes** **

**Figure 2:**
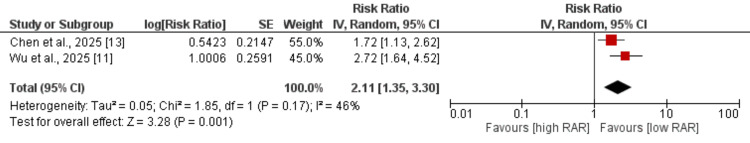
Effect of high RAR on mortality. Sources: References [11,13]. RAR: red blood cell distribution width-to-albumin ratio.

Effect of RDW/Alb on mortality in acute pancreatitis patients: Figure 2 displays the findings of two studies that evaluated the impact of RDW/Alb on mortality. High RDW/Alb was linked to a higher risk of death, according to pooled analysis (RR: 2.11, 95% CI: 1.35-3.30). The study results showed moderate heterogeneity (I²: 46%). 

**Figure 3:**
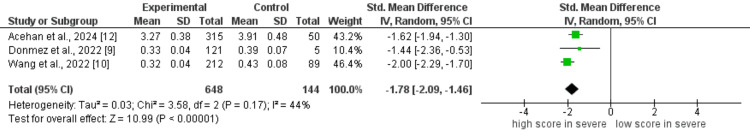
Effect of high RAR on severity. Sources: References [9-10,12]. RAR: red blood cell distribution width-to-albumin ratio.

Effect of RDW/Alb on severity in acute pancreatitis patients: We compared mean values of RDW/Alb on the severity of acute pancreatitis, and the results are demonstrated in Figure 3. Pooled analysis showed that mean RDW/Alb was significantly lower in patients with mild acute pancreatitis compared to patients with severe acute pancreatitis (mean difference (MD): -1.78, 95% CI: -2.09 to -1.46). Moderate heterogeneity was reported among the study results (I²: 44%). 

Discussion 

This meta-analysis assessed the prognostic value of the RDW/Alb ratio in acute pancreatitis (AP). RDW/Alb, a novel biomarker derived from red cell distribution width (RDW) and albumin (Alb), has been linked to disease severity and mortality in patients with AP. However, to date, no systematic review or meta-analysis has been conducted to evaluate the independent predictive value of RDW/Alb for AP prognosis. To the best of our knowledge, this is the first meta-analysis to explore the association between RDW/Alb and mortality in patients with acute pancreatitis. 

In recent years, various diagnostic and prognostic markers, both individually and in combination, have been evaluated in patients with acute pancreatitis (AP) [14,15]. Since the identification of elevated RDW as a predictor of poor outcomes in chronic heart failure in 2007, numerous studies have demonstrated its significant association with short-term mortality outcomes in various inflammatory diseases [16]. RDW, which is calculated based on the mean red blood cell volume and its standard deviation, is a commonly used erythrocyte parameter that can be quickly and easily obtained through routine blood tests [17]. Its low cost and minimal technical requirements make it readily available in most healthcare facilities. Traditionally, RDW has been considered a useful marker for differentiating between thalassemia and iron deficiency anemia [17]. 

The development of acute pancreatitis (AP) is closely linked to oxidative stress and the activation of the inflammatory response, both of which can contribute to tissue injury. In contrast, serum albumin facilitates the production of various anti-inflammatory agents, such as lipoproteins and lysins, which support tissue repair and help resolve inflammation [18]. As a result, significant albumin consumption occurs during this process, which may partly explain the association between low albumin levels and poor outcomes in AP. However, serum albumin levels are influenced by various physiological and pathological factors, including chronic illnesses, nutritional status, and systemic inflammation, which may reduce the reliability of a single measurement as a predictor [19,20]. A study by Chen et al. demonstrated that albumin alone had a low area under the curve (AUC) for predicting all-cause mortality in AP. However, when the ratio of RDW to albumin (RAR) was assessed in the same study, it showed a higher AUC than albumin alone, as the ratio reflects the opposing changes caused by the two different factors, thereby minimizing the impact of individual variations on the predictive mechanism [13]. The study also reported that RAR had a higher AUC compared to the Sequential Organ Failure Assessment (SOFA) score [12]. Similarly, Donmez et al. [9] found that RAR values differed significantly among patients with varying severities of acute biliary pancreatitis, with the highest RAR observed in those with severe acute pancreatitis. They also identified a dose-response relationship between elevated RAR and increased mortality risk. 

Acehan et al. explored the connection between RAR and both in-hospital mortality and disease severity in individuals with acute pancreatitis (AP). Their findings indicated that RAR measured 48 hours post-admission served as an independent predictor of severe acute pancreatitis (SAP), with a threshold value of 4.35. Regarding in-hospital mortality, the AUC for RAR at 48 hours was 0.960 (95% CI: 0.931-0.989), which was significantly higher than the AUC values of established scoring models [12]. Similarly, Hidalgo et al. [21] assessed the predictive utility of the Charlson and Elixhauser comorbidity indices for early mortality in pancreatitis patients in a cohort of 110,021 individuals. The AUCs for these indices were 0.633 (95% CI: 0.623-0.641) and 0.666 (95% CI: 0.657-0.674), respectively. In the present study, the AUC for RAR was 0.669 (95% CI: 0.617-0.720), surpassing the Charlson and Elixhauser indices and exceeding the AUC of 0.651 (95% CI: 0.597-0.701) reported by Efgan et al. for Prognostic Nutritional Index (PNI) [22]. This implies that RAR may serve as a more effective early mortality predictor in acute pancreatitis. Although RAR exhibits a comparatively lower AUC than conventional scoring tools like the Ranson and Bedside Index of Severity in Acute Pancreatitis (BISAP) systems [23], it offers notable benefits in terms of rapid assessment and cost efficiency. 

The present meta-analysis has certain limitations. First, only five studies were included, and outcomes assessed in this meta-analysis, including mortality and severity, were assessed by only two and three studies, respectively. Secondly, all included studies were retrospective, which is associated with inevitable biases, which may affect the authenticity of the results. Additionally, the included studies did not include enough related information on treatment, which would have allowed for a more comprehensive evaluation of patient outcomes. We were not able to perform subgroup analysis based on the underlying comorbidities and so on. There is high variation in the study setting as some studies were performed in ICU, and some included ICU as well as non-ICU patients. Due to the unavailability of data, we were not able to perform a subgroup analysis. Further prospective studies with larger sample sizes are needed to validate these findings and establish optimal RAR cutoff values for clinical decision-making in acute pancreatitis.

## Conclusions

This systematic review and meta-analysis demonstrates that elevated red blood cell distribution width-to-albumin ratio (RAR) is significantly associated with increased mortality and severity in patients with acute pancreatitis. As a simple, readily available, and cost-effective biomarker derived from routine blood tests, RAR shows promise as a valuable prognostic tool for early risk stratification in acute pancreatitis management. While RAR may not match the discriminative power of conventional scoring systems, its accessibility and rapid assessment capability offer distinct advantages in resource-limited settings and emergency situations. However, the limitations of our analysis, including the small number of included studies and their retrospective nature, necessitate cautious interpretation. Future large-scale, prospective studies are needed to validate these findings and establish optimal cutoff values for RAR in different clinical contexts and patient populations.
